# Gene Expression Profiling in Viable but Nonculturable (VBNC) Cells of *Pseudomonas syringae* pv. *syringae*

**DOI:** 10.3389/fmicb.2015.01419

**Published:** 2015-12-18

**Authors:** Olga A. Postnikova, Jonathan Shao, Norton M. Mock, Con J. Baker, Lev G. Nemchinov

**Affiliations:** Molecular Plant Pathology Laboratory, Beltsville Agricultural Research Center, United States Department of Agriculture, Agricultural Research ServiceBeltsville, MD, USA

**Keywords:** VBNC, plant pathogenic bacteria, *P. syringae* pv. *syringae*, RNA-seq, global gene expression profiling

## Abstract

*Pseudomonas syringae* infects diverse crop plants and comprises at least 50 different pathovar strains with different host ranges. More information on the physiological and molecular effects of the host inhibitory environment on the pathogen is needed to develop resistant cultivars. Recently, we reported an *in vitro* model system that mimics the redox pulse associated with the oxidative burst in plant cells inoculated with *Pseudomonas syringae* pv. *syringae*. Using this system, we demonstrated that oxidation of acetosyringone, a major extracellular phenolic compound induced in some plants in response to bacteria, rendered *Pseudomonas syringae* pv. *syringae* to a “viable but nonculturable” (VBNC) state. Here we performed a large scale transcriptome profiling of *P. s*. pv. *syringae* in the VBNC state induced by acetosyringone treatment and identified bacterial genes and pathways presumably associated with this condition. The findings offer insight into what events occur when bacterial pathogens are first encountered and host defense responses are triggered. The acquired knowledge will improve our understanding of the molecular mechanisms of stress tolerance. We believe that this is the first work on global gene expression profiling of VBNC cells in plant pathogenic bacteria.

## Introduction

*Pseudomonas syringae* infects many crop plants and is also widely spread in non-agricultural niches (Morris et al., 2008, 2013). At least 50 different pathovar strains have been described based on pathogenicity toward different hosts (https://microbewiki.kenyon.edu/index.php/Pseudomonas_syringae; http://pseudomonas-syringae.org/). It is one of the most studied bacterial pathogens. Three complete annotated genomes of *P. syringae* (*P. syringae* pv. *syringae* B728a, *P. syringae* pv. *tomato* DC3000, and *P. syringae* pv. *phaseolicola* 1448A) and multiple draft genome sequences are currently available (Buell et al., 2003; Creasy et al., 2005; Feil et al., 2005; Joardar et al., 2005; Baltrus et al., 2011).

One of our objectives is related to understanding molecular mechanisms of stress tolerance in alfalfa (*Medicago sativa*), the most widely grown forage crop in the world (Postnikova et al., 2013, 2015). It was recently reported that bacterial stem blight of alfalfa caused by *P. syringae* pv. *syringae* ALF3 occurs sporadically in the central and western U.S. and yield losses from the pathogen can be as high as 50% of the first harvest (Samac et al., 2014). More information on the physiological and molecular events of the host inhibitory environment on the pathogen is needed to develop resistant cultivars.

Mechanisms of the bacterial pathogenicity and host responses to infection have been studied in great details (Jones and Dangl, 2006; Cunnac et al., 2009; Baltrus et al., 2011). Production of reactive oxygen species (ROS) seem to be at the core of the complex signaling network initiated upon pathogen invasion (O'Brien et al., 2012). During interactions with avirulent pathogens, generation of ROS precedes hypersensitive cell death response (HR) in the resistant host (Zurbriggen et al., 2009). Resistant (*R*) gene-mediated recognition of a pathogen's Avr protein (a product of a pathogen's *Avirulence* gene) leads to ROS via poorly understood mechanisms (Mur et al., 2008). Throughout the evolution, plant cells have evolved to overcome damaging effects by balancing ROS production and scavenging and by using ROS as signal molecules in HR pathways (Bailey-Serres and Mittler, 2006; Mur et al., 2008; Zurbriggen et al., 2009). Key enzymes implicated in ROS production during resistant host-pathogen interactions are NADH oxidases that generate superoxide (${\text{O}}_{2}^{-}$) and cell-wall peroxidases, a source of hydrogen peroxide (H_2_O_2_) (Torres et al., 2006).

Plant phenolic compounds are well-known reductants of peroxidases and scavengers of ROS (Blokhina et al., 2003). One of naturally occurring extracellular phenols, acetosyringone, has bioactive characteristics that can influence early phases of plant-pathogen interactions possibly by sharply increasing the redox potential and thus creating a local environment hostile to pathogen ingress (Baker et al., 2005, 2014). Oxidation of acetosyringone produces a relatively stable intermediate radical that appears to be responsible for the high increase in redox potential. These conditions also induce pathogenic bacteria into a “viable but nonculturable state” (VBNC) (Mock et al., 2015). The VBNC state is considered to be a possible survival strategy for bacteria in response to various stresses and as such has numerous implications—most importantly, renewed ability to cause infection (Oliver, 2010). Continuous gene expression in VBNC cells indicates that they remain metabolically functional (Oliver, 2010; Meng et al., 2015).

In this work, we performed gene expression profiling in *P. s*. pv. *syringae* cells rendered VBNC by exposure to the oxidation of acetosyringone and identified bacterial genes and pathways presumably associated with this condition. The findings offer insight into what might happen in the plant when bacterial pathogens are first encountered and host defense responses are triggered. The acquired knowledge will improve our understanding of the molecular mechanisms of stress tolerance, biological relevance of VBNC and microbial dormancy as a means to overcome unfavorable environmental conditions. This information may potentially be applied to a wide range of crop plants infected by *P. syringae*, including our target crop, *M. sativa*.

## Materials and methods

### Chemicals

The LIVE/DEAD® BacLight fluorescent stain used for microscopy was obtained from Life Technologies (Grand Island, NY). All other chemicals were obtained from Sigma Aldrich Chemicals, Inc (St. Louis, MO). Stock solutions of acetosyringone (3,5-dimethoxy-4- hydroxyacetophenone), 10 mM; horseradish peroxidase (P8375), 720 U ml^−1^; hydrogen peroxide, 10 mM, were prepared in deionized water.

### Bacteria

*P. syringae* pv. *syringae* 61 was prepared as previously described (Baker et al., 2011). It was routinely maintained on King's B media augmented with 25 μg/ml naladixic acid. Long term storage of the bacteria was in the same media supplemented with 15% glycerol in liquid nitrogen. For experiments, bacteria were grown for 18–20 h in King's B broth (augmented with naladixic acid) in dark, at 190 rpm, 30°C. Overnight cultures were centrifuged at 8000 rpm for 3 min at room temperature in a fixed rotor. Pellets were washed with deionized water, centrifuged, and then suspended in a small amount of deionized water to various concentrations. The bacterial concentration of this slurry was estimated by diluting aliquots and using optical density (0.1 ODU_600nm_ ≈ 10^8^ bacteria/ml). The final concentration was adjusted to about 10 ODU so that addition of about 200 μL of the bacterial suspension to 20 ml of reaction mixture would result in the final concentration of 1 × 10^8^ CFU ml^−1^.

To determine colony forming units (CFU) serial dilutions were made in sterile deionized water, and plated on King's B agar. A series of dilutions consisting of 1:5 then 1:2 were made and found to give better accuracy than traditional 1:10 dilutions. For each sample, two replications, 10 μl, of 7 dilutions were placed on a single plate (14 spots/plate). The agar plates were incubated at 30°C for 24–48 h until colonies were large enough to count. Dilutions which produced between 5 and 30 colonies within the ~7 mm diameter spot were considered optimal for determining CFU (Baker et al., 2011).

### Monitoring redox potential *in vitro*

As previously described (Mock et al., 2015), reaction mixtures were contained in a 50 ml plastic beaker in a temperature controlled shaking water bath, 190 rpm, 27°C. The routine reaction mixture, 20 ml, contained 10 mM KH_2_PO_4_ buffer, pH 6.0, 100 μM acetosyringone, 100 μM H_2_O_2_, and 0.72 U/ml horseradish peroxidase. The acetosyringone and H_2_O_2_ concentrations were always equimolar. A combination redox electrode, platinum wire with an internal Ag/AgCl (saturated) reference (Microelectrodes Inc., Londonderry, NH, USA), was placed in the reaction mixture and the open-circuit potential read every 0.25 min using a PCIe-6363 data acquisition board and LabVIEW software (National Instruments Corp., Austin, TX, USA) on a Windows 7 operating system.

### Live/dead fluorescent stain for bacterial viability using microscopy

Samples of the reaction mixture plus bacteria, 1 ml, were treated with 1 μl of LIVE/DEAD® BacLight fluorescent stain. After 15 min 10 μL was applied to a slide with coverslip and viewed with a Zeiss Axioskop fluorescent microscope (Carl Zeiss International). The two color fluorescence assay uses green nucleic acid stain that penetrates both live and dead cells, while the red propidium iodide stain is excluded from live cells; neither dye was affected by the reaction components. Pictures were obtained from a Confocal Laser Scanning Microscope Zeiss 710.

### RNA extraction, first-strand synthesis, and quantitative real-time PCR

RNA was extracted and purified using Qiagen RNeasy mini kit as described by manufacturer (Qiagen). Amplification was conducted with a Rotor Gene Q real time PCR cycler (Qiagen) using Rotor-Gene SYBR® Green PCR kit (Qiagen) in four biological and two technical replicas using the following parameters: 95°C/10 min (one cycle), 95°C/10 s, and 60°C/45 s (40 cycles). cDNA for qPCRs was made with random hexamers from the same RNA samples that were used for RNA-sequencing. 16S rRNA gene of *P. s*. pv. *syringae* B728a (NC_007005) was used as a reference in all qPCR experiments. The specificity of all amplifications was confirmed by single-peak melting curves. Delta Delta C (T) method ($2_{\text{T}}^{-\Delta \Delta \text{C}}$) was used for analysis of relative expression. To obtain a final ratio for any given gene, an average and a standard deviation (SD) for all biological replicates were calculated.

### RNA-Seq and read counts

RNA-sequencing was performed by Next Generation Sequencing Center, John Hopkins University (Baltimore, MD, USA) for a fee using the Illumina HiSeq 2000 system. There were four replicates for each experimental condition and for untreated control. cDNA libraries were generated using rRNA depletion method. The strand-specific paired-end-reads were mapped onto the reference genome *P. syringae* pv. *syringae B728a* (NC_007005.1) using the strand specific option forward and reverse of the RNAseq analysis module in the CLC Genomic Workbench 8.0.2 software (http://www.clcbio.com/products/clc-main-workbench/). The software maps reads only in their forward or reverse orientation, depending on the option selected. The strand specific option in the CLC workbench software allows the assignment of reads to the correct gene in cases where overlapping genes are located on different strands. The forward option measures the degree of sense transcription and the reverse option measures the degree of anti-sense transcription. The DESeq 2 package from Bioconductor was used to estimate sample quality (PCA) and expression level of the genes (Anders and Huber, 2010). Genes with fold change more than 2 and false discovery rate (FDR) < 0.025 were counted as a differentially expressed. Analysis of the number of reads for each gene mapped on sense or antisense strand showed that 241 genes had at least 2 times more reads mapped on the their opposite strand, according to *P. syringae* pv. *syringae B728a* genome annotation. These genes were excluded from further analysis, because orientation of the expression (sense or antisense) could not be accurately determined based on the reference genome. The Pseudomonas Genome Database (Winsor et al., 2011) and website (http://genome2d.molgenrug.nl/) were used for gene search and functional analysis.

### Gene representation analysis

We took advantage of the previously published functional categorization described by Yu et al. (2013). Customer R script for Fisher's exact test was used to evaluate overrepresentation of the differentially expressed genes (DEGs). The test was performed separately for the induced and repressed genes.

## Results

### Acetosyringone causes nonculturability of *P. syringae* pv. *syringae*

**Figure 1:**
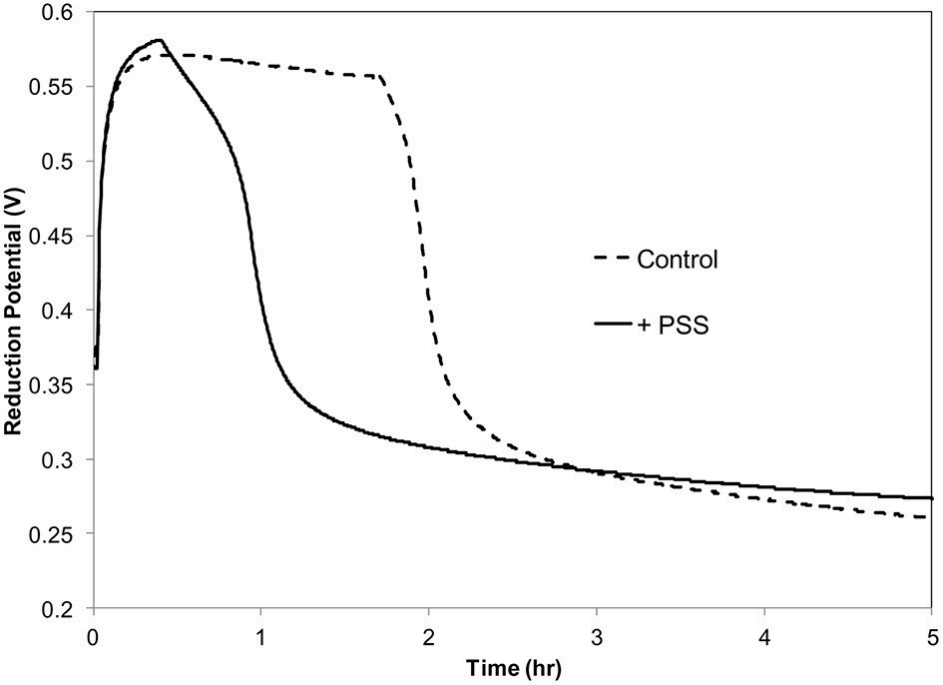
**Redox potential of acetosyringone oxidation with and without ***P. syringae*** pv. ***syringae*****. Reaction contains: 100 μM acetosyringone, 100 μM H_2_O_2_ and 0.72 U ml ^−1^ horseradish peroxidase in 10 mM KH_2_PO_4_, pH 6.

Previously it was demonstrated that *in vitro* oxidation of acetosyringone with hydrogen peroxide can create a prolonged oxidative environment mimicking the oxidative burst in tobacco suspension cells inoculated with *P. s*. pv. *syringae* 61 (Mock et al., 2015). It was also demonstrated that these conditions induced a VBNC-like state in the bacteria. In order to collect sufficient amounts of RNA for this study, we used a higher bacterial concentration,10^8^ CFU ml^−1^, than used previously. *P. s*. pv. *syringae* 61 was incubated with 100 μM H_2_O_2_, 100 μM acetosyringone (ACE) and 0.72 Uml^−1^ peroxidase (POX) in 10 mM KH_2_PO_4_ pH 6.0 at 27°C. The period of the oxidative burst was shortened by an hour due to the increased scavenging of H_2_O_2_ by the high concentration of bacteria (Figure 1).

**Figure 2:**
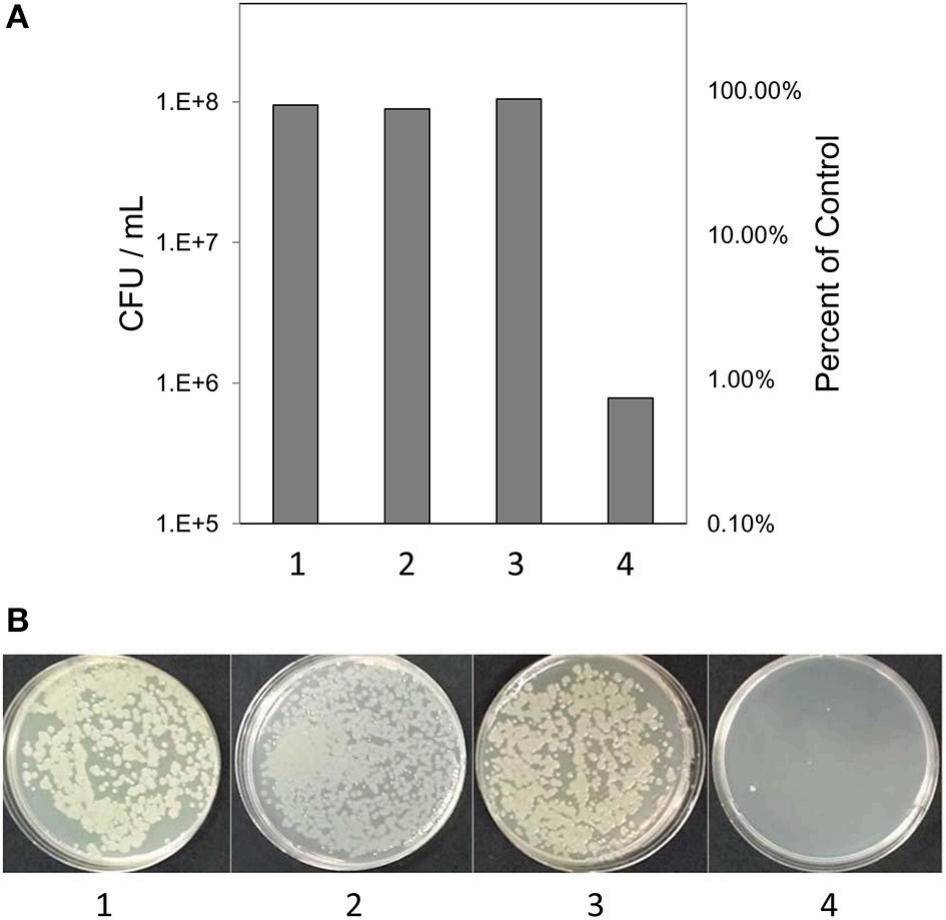
**(A)** Culturability of the ***of P. syringae*** pv. ***syringae*** after incubation in different reaction mixtures. **(B)** Visual comparison of culture plates after incubation of bacteria in different reaction mixtures. (1) 10 mM KH_2_PO4, pH 6.0; (2) H_2_O_2_ and 10 mM KH_2_PO_4_; (3) 10 mM KH_2_PO_4_, 100 μM H_2_O_2_ and 0.72 U ml ^−1^ horseradish peroxidase; (4) 100 μM acetosyringone, 100 μM H_2_O_2_ and 0.72 U ml ^−1^ horseradish peroxidase.

**Figure 3:**
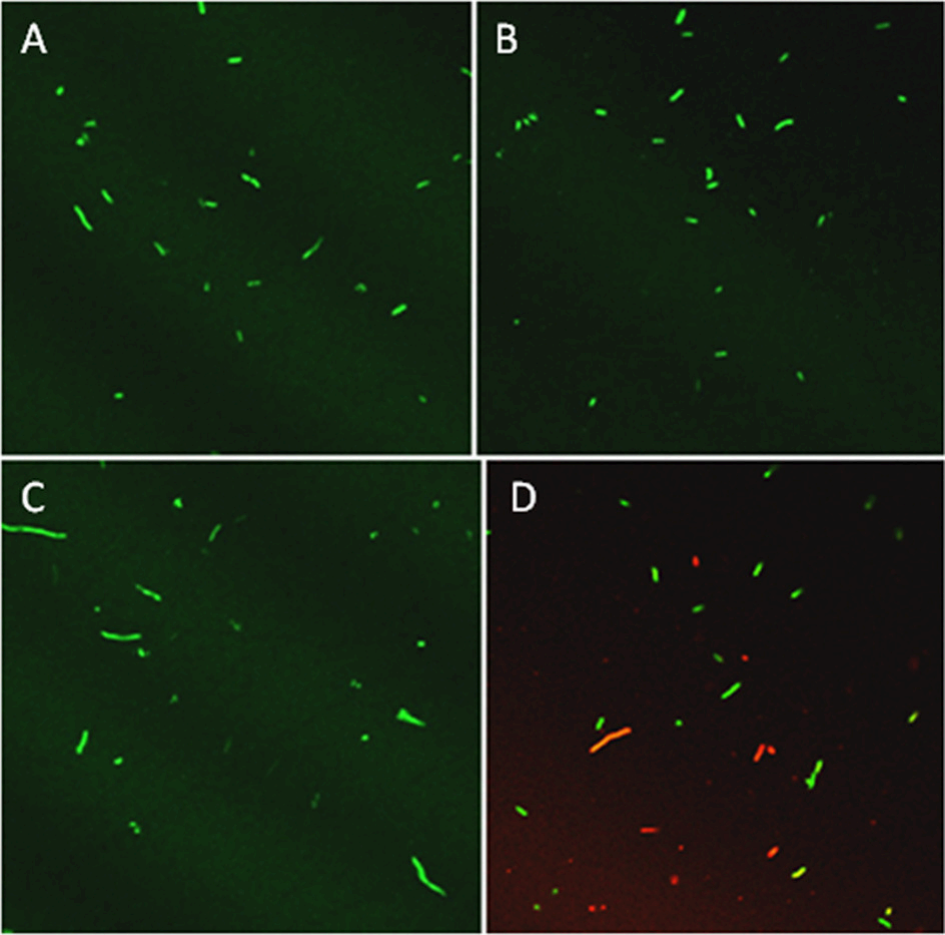
**Confocal imaging of ***P. syringae*** pv. ***syringae*** treated with LIVE/DEAD® BacLight fluorescent stain**. **(A)** Control, 3 h incubation in 10 mM KH_2_PO_4_, pH 6.0. **(B)** Three hours incubation in 100 μM acetosyringone, 100 μM H_2_O_2_ and 0.72 U ml ^−1^ horseradish peroxidase. **(C)** Control, 48 h incubation in 10 mM KH_2_PO4, pH 6.0. **(D)** Reaction, 48 h incubation in 100 μM acetosyringone, 100 μM H_2_O_2_ and 0.72 U ml ^−1^ horseradish peroxidase.

Based on previous work with this system (Mock et al., 2015), after 3 h samples were tested for culturability and viability; the redox potential was stabilized and reproduction by any surviving bacteria would be minimal. Culturability was reduced by 99% based on dilution plating (Figure 2A). Culture plates of samples equally diluted (10^5^) are shown for visual comparison (Figure 2B). Viability of 3 h-treated bacterial cells was determined by fluorescent staining with the LIVE/DEAD BacLight stain (Thermo Fisher Scientific, Inc.) Cells with intact membranes block the red stain. Both controls and treated bacterial cells fluoresced green (Figures 3A,B), which means that cell death did not occur in nonculturable bacteria and they maintained membrane integrity after treatment with ACE, POX, and H_2_O_2_. Only after 48 h did treated cells begin to take up the red stain. The control cells did not (Figures 3C,D).

### Categorization of RNA-Seq reads and antisense activity

**Figure 4:**
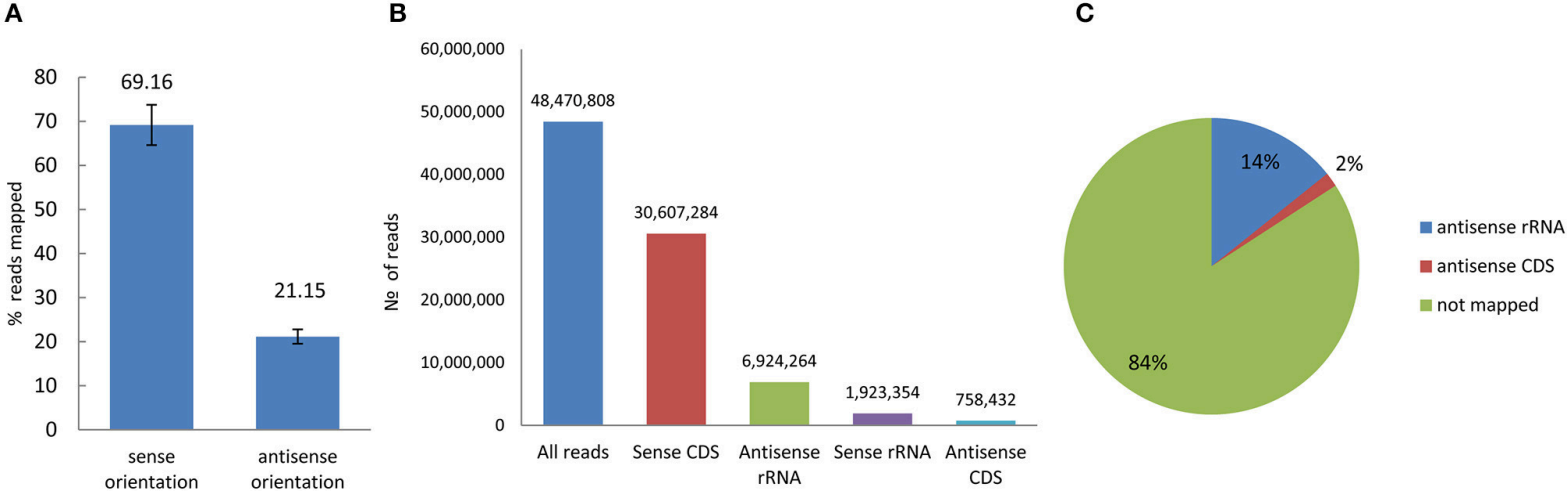
**Reads mapped to the reference genome of ***P. syringae*** pv. ***syringae*** B728a in sense and antisense orientation. (A)** Percentage of overall alignment rate (averaged for pare-end reads obtained from control and VBNC samples). **(B)** Distribution of the average number of the mapped reads (eight replicates) in sense and antisense orientation **(C)** Characterization of the antisense transcripts.

The amount of total RNA extracted from the bacterial control and the bacteria treated with H_2_O_2_, POX, and ACE (VBNC) was not significantly different (not shown). Four control replicates and four treated replicates were generated and a total of 193,883,230 short reads were obtained from the eight strand-specific ribosomal RNA-depleted libraries. The number of reads mapped to the reference genome from the four control libraries was 45,265,084 and the number of reads mapped to the reference genome from the four VBNC libraries was 47,162,193. The strand-specific paired-end-reads were mapped using the RNAseq analysis module in the CLC Genomic Workbench 8.0.2. Overall alignment rate was on average 69% for sense and 21% for antisense transcription (Figure 4). Among non-rRNA-derived short reads 2% were mapped to the coding sequences (CDS) in the antisense orientation. These reads were distributed between 2455 genes of the control and 1776 genes of the VBNC libraries (cut off = 100 reads per CDS region) (Figure 4). Antisense transcription plays an important role in regulation of gene expression (Pelechano and Steinmetz, 2013) and reduction of genic antisense RNAs in the VBNC cells might have implications or correlate with amount of transcripts derived from the sense strand.

A large number of reads in both libraries (averaged 36%) originated from two housekeeping small RNAs, rnpB and SsrA, encoding Ribonuclease P, and transfer-messenger RNA, respectively (Hershberg et al., 2003). Since the proportion of these reads in each of the eight replicates was the same, it did not affect differential expression analysis of other genes by DESeq 2. This fact was additionally confirmed by removal of the rnpB and SsrA reads from all libraries and re-run of the DESe 2 software with the remaining reads. The expression level did not change. To double check if abundance of rnpB and SsrA sRNAs is common in other experiments, we extracted raw RNA-seq data similarly obtained elsewhere on *P. syringae* from the Sequence Read Archive (SRA, NCBI). In the random example used for comparison (not shown), these sRNAs were abundantly expressed at least at the same level as in this experiment.

### Basal gene expression in untreated nutrient-deprived bacteria

**Figure 5:**
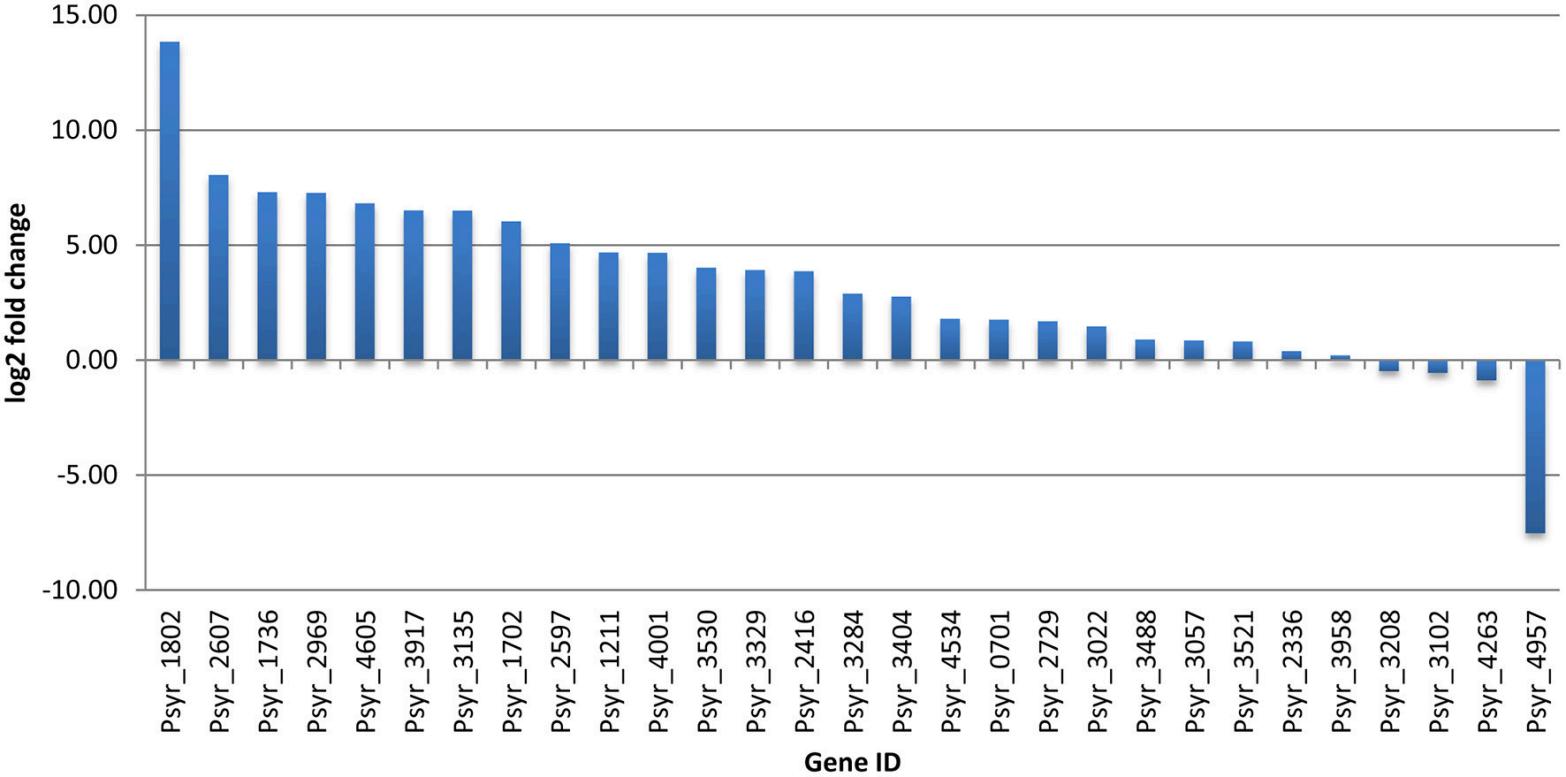
**Comparison of gene expression during the log phase of bacterial growth in King's B media and in nutrient-deprived bacteria incubated for 3 h in 10 mM KH_2_PO_4_, pH 6.0 (ratio)**. Data are based on qPCR results.

Overnight cultures of bacterial cells in King's B media were centrifuged, washed and resuspended in a buffer prior to the treatment with acetosyringone. Since measurements were taken at 3 h after incubation in the reaction mixture, *P. s*. pv. *syringae* cells were nutrition- deprived for the respective amount of time. To assess a relative contribution of nutrient deprivation stress to the gene expression levels in untreated bacteria, we performed quantitative real-time PCR (qPCR) with 29 genes. The genes were selected based on the preliminary analysis of the RNA-seq data. QPCR data were normalized against the 16S rRNA housekeeping gene. Basal gene expression of 86% of the selected genes was significantly decreased (at least two-fold, in some genes more than a 1000 fold) as compared to the log phase bacteria grown in King's B media (Figure 5). This suggested that nutrition-deprived bacteria kept its expression level to a minimum. Nevertheless, untreated *P. s*. pv. *syringae* were not rendered VBNC (Figure 2). Since the subsequent exposure of bacteria to the oxidative burst happened at the background of non-optimal growth conditions, we considered starvation stress a potentially important factor that may have contributed to changes of gene expression during the VBNC state.

### Bacterial gene expression after 3 h of exposure to acetosyringone oxidation

We found 929 DEGs after bacteria were incubated for a period of 3 h in the reaction mixtures containing 100 μM ACE, POX, and H_2_O_2_ (Supplementary Table 1). The majority of them (606 DEGs) were down-regulated and about one-third (323 DEGs) were induced. QPCR was performed with arbitrarily selected 50 genes classified as differentially expressed based on the transcriptomic analysis. QPCR results showed a strong correlation with transcriptomic data (Pearson correlation coefficient *r* = 0.76, Supplementary Table 2). Ten percents of the checked genes (5 out of 50) did not match between RNA-seq and qPCR data, which could be due to the sequence differences between the reference genome used for reads mapping and the genome of *P. s*. pv. *syringae* 61 used in this study.

#### Overrepresented functional categories

**Figure 6:**
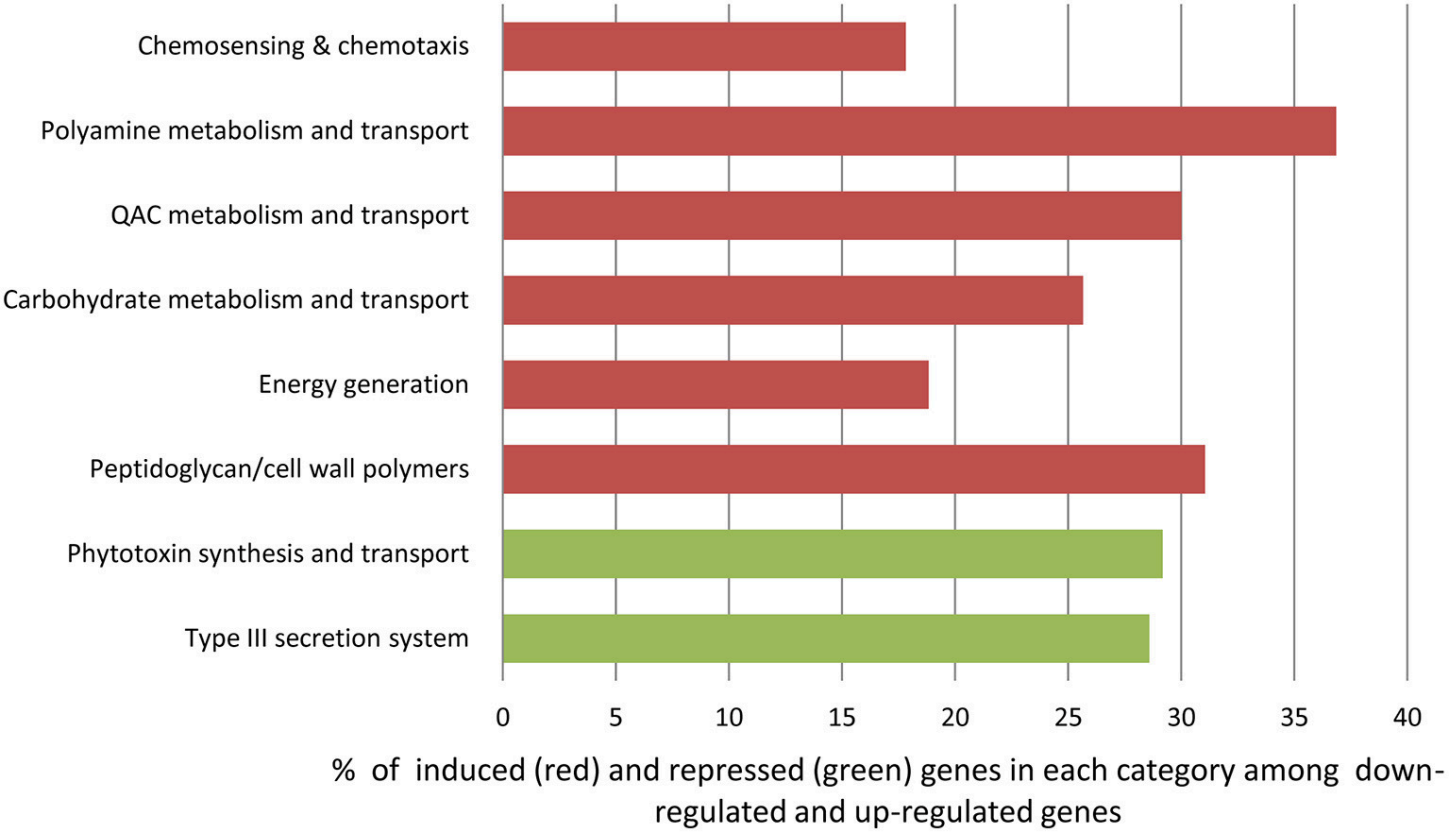
**Overrepresented functional categories identified in VBNC cells using gene representation analysis (Yu et al., 2013)**.

Using gene representation analysis essentially as described by Yu et al. (2013) for the reference genome *P. s*. pv. *syringae* B728a, all DEGs were assigned to functional categories for annotation and description of their prospective biological functions. In our experience, this classification provided more detailed, organism-specific, and less confusing categorization as compared to the GO annotation that often resulted in multiple inconsistent interpretations. Categories *Carbohydrate metabolism and transport, QAC metabolism and transport* (quaternary ammonium compounds), *Polyamine metabolisms, Chemosensing & chemotaxis, Energy generation*, and *Peptidoglycan/cell wall polymers* were overrepresented among up-regulated genes at the 3 h time point (Figure 6 and Supplementary Table 3). The most significantly up-regulated genes in each of these categories are shown in Table 1. Category *Energy generation* contains a block of 10 up-regulated genes encoding different subunits of NADH dehydrogenase (ubiquinone) (catalyses the transfer of electrons from NADH to coenzyme Q10), which indicates that processes involved in detoxification of ROS are activated in the VBNC mode (Table 1). Gene encoding aconitate hydratase or aconitase (Psyr_3404) from the same category (*Energy generation*) is also up-regulated (Table 1). Aconitase is an iron-sulfur protein, which catalyses the reversible isomerization of citrate and isocitrate via *cis* aconitate (Prodromou et al., 1991; Tang et al., 2002). The iron-sulfur cluster of the aconitase is highly sensitive to oxidation by superoxide (Gardner, 2002). Induction of a major facilitator transporter (Psyr_3138) and several ABC transporters in the categories *Carbohydrate metabolism and transport* (Psyr_3264, Psyr_2437, Psyr_1737, and Psyr_1738) and *Polyamine metabolism and transport* (Psyr_4862 and Psyr_4863) suggests that cellular systems implicated in translocation of different substrates across membranes, such as uptake of nutrients or efflux of toxins, remain operational. One of the up-regulated ABC transporters, Psyr_3265 represents periplasmic binding proteins, which are the primary receptors for chemotaxis and mediate solute uptake. Polyamines are required for growth of bacterial cells. In *Escherichia coli*, approximately half of the polyamine-associated genes encode enzymes involved in their biosynthesis or degradation, and the rest are responsible for polyamine transport (Igarashi and Kashiwagi, 1999). Majority of the up-regulated genes in the category *Polyamine metabolism and transport* (7 out of 9) are transport-related, which implies polyamine homeostasis is important for VBNC cells. Functional categories *Type III secretion system* and *Phytotoxin synthesis and transport* were overrepresented among down-regulated genes, suggesting minor roles of the pathogenesis-related genes in the processes associated with the VBNC state.

**Table 1:** **The most significantly up-regulated genes in each of the overrepresented functional categories**.

	**Log_2_ fold change**	**Annotation**	**Category**
Psyr_3265	3.50	Periplasmic binding protein	Carbohydrate metabolism and transport
Psyr_3138	3.97	Major facilitator transporter	Carbohydrate metabolism and transport
Psyr_2440	3.21	Extracellular solute-binding protein	Carbohydrate metabolism and transport
Psyr_1737	3.71	ABC transporter	Carbohydrate metabolism and transport
Psyr_1738	3.24	ABC transporter	Carbohydrate metabolism and transport
Psyr_2356	1.95	Histidine kinase, HAMP	Chemosensing & chemotaxis
Psyr_0905	1.73	Histidine kinase, HAMP	Chemosensing & chemotaxis
Psyr_2215	1.65	CheW-like protein	Chemosensing & chemotaxis
Psyr_3404	1.87	acnA, aconitate hydratase	Energy generation
Psyr_3204	1.02	NADH dehydrogenase subunit I	Energy generation
Psyr_3201	1.03	NADH dehydrogenase I subunit F	Energy generation
Psyr_3198	1.08	NADH dehydrogenase subunit B	Energy generation
Psyr_3202	1.10	NADH dehydrogenase subunit G	Energy generation
Psyr_3200	1.11	NADH dehydrogenase subunit E	Energy generation
Psyr_3203	1.21	NADH dehydrogenase subunit H	Energy generation
Psyr_3207	1.26	NADH dehydrogenase subunit L	Energy generation
Psyr_3205	1.27	NADH dehydrogenase subunit J	Energy generation
Psyr_3208	1.34	NADH dehydrogenase subunit M	Energy generation
Psyr_3209	1.35	NADH dehydrogenase subunit N	Energy generation
Psyr_2414	2.10	pbpG,D-alanyl-D-alanine endopeptidase	Peptidoglycan/cell wall polymers
Psyr_4102	1.22	murG	Peptidoglycan/cell wall polymers
Psyr_4863	1.84	Spermidine/putrescine ABC transporter	Polyamine metabolism and transport
Psyr_4862	2.15	Binding-protein dependent transport system	Polyamine metabolism and transport
Psyr_4781	2.43	4Fe-4S ferredoxin	QAC metabolism and transport
Psyr_4711	2.49	Glycine betaine/L-proline transporter	QAC metabolism and transport

Nine up-regulated genes from the category *Peptidoglycan/cell wall polymers* (Supplementary Tables 1, 3), including D-alanyl-D-alanine endopeptidase (Psyr_2414) and UDPdiphospho-muramoylpentapeptide beta-N- acetylglucosaminyltransferase (Psyr_4102, *Mur*G) are involved in the cell wall formation and may play a specialized role in remodeling the cell wall (http://www.uniprot.org/uniprot/P0AFI5). Genes assigned to the overrepresented functional category QAC *metabolism and transport* may take part in the biosynthesis of QAC osmoptotectants in bacteria as well as in biodegradation/metabolism of exogenic QAC. QAC are salts of quaternary ammonium cations and are known for their antimicrobial activity. One of the up-regulated genes in this category is a transporter for glycine betaine/L-proline (Psyr_4711). Glycine betaine acts as an osmoprotectant in plants, bacteria, and animals and its accumulation can reduce effect of stress and increase survival (Hanson et al., 1994). Induction of 4Fe-4S ferredoxin (Psyr_4781) suggests activation of electron transfer pathways in different metabolic reactions. Up-regulation of flavin reductase in the same category (Psyr_4782) indicates that a variety of processes associated with the reduction of flavins in VBNC cells are operational.

#### Other functional categories

If the proportion of input genes in the particular category is small, the resulting functional categories will not be overrepresented. However, altered expression of individual genes in these categories may be of interest because they can be an integral part of the larger gene network contributing to the VBNC state. Gene representation analysis (Yu et al., 2013) assigned 386 DEGs to 54 functional categories that were not significantly overrepresented (Supplementary Table 2). A number of genes involved in *Amino acid metabolism and transport* were differentially expressed and among them 17 were up-regulated (Supplementary Tables 1, 3). Induced and repressed genes in this category encode enzymes participating in valine, leucine and isoleucine biosynthesis and degradation pathways, glycine, serine and threonine metabolism, arginin biosynthesis, and alanin, aspirate and glutamate metabolism. Since genes encoding amino acid synthesis enzymes are stringently regulated depending on the bacterial demands, their products must be critical for the control of biosynthesis and degradation of the amino acids essential for transition to VBNC mode. A group of six genes in the category *Signal transduction mechanisms* was up-regulated (Supplementary Tables 1, 3). These genes are classified as response regulator receivers involved in a phosphorelay signal transduction, a two-component regulatory system, consisting of histitine kinases activated by a wide range of extracellular signals and response regulators that mediate the cellular response (Loomis et al., 1997). Up-regulation of genes encoding response regulator receiver (Psyr_3709), sensor histidine kinase (Psyr_3708), and carbon starvation protein (Psyr_4273) in the category *Stress resistance* indicates their potential involvement in the change in state and activity of bacteria as a result of acetosyringone oxidation. Activation of DNA repair processes (category *Replication and DNA repair*, Psyr_1259, Psyr_4703, Psyr_0001, and Psyr_4640) means that VBNC cells are able to retain oxidative DNA damage. Three highly induced genes in the category *Secretion/Efflux/Export* (Psyr_2967, Psyr_2968, and Psyr_69) are part of the operon of the multidrug resistance, efflux pump MexEF-OprN. The efflux pumps not only confer resistance to antibiotics, but have roles in bacterial pathogenicity and resistance to host-defense molecules (Piddock, 2006).

#### Transcription factors (TFs)

Forty TFs differentially changed their expression after 3 h treatment period; 11 of them were up-regulated (Table 2 and Supplementary Table 1). Notably, among up-regulated TFs were MarR and LysR proteins. MarR TFs, multiple antibiotic resistance regulators and members of the winged helix-turn-helix TF family, regulate activity of genes involved in responses to many environmental factors, including oxidative stress and degradation of phenolic compounds (Grove, 2013). LysR TFs are the most abundant transcriptional regulators in bacteria that govern a diverse set of genes, including those involved in virulence, metabolism, quorum sensing, and motility (Maddocks and Oyston, 2008). One of the induced TFs (ID Psyr_3022) is a member of the Lrp/AsnC (leucine-responsive regulatory protein/asparagine synthase C products) family of transcriptional regulators, widespread in bacteria. Activated TF Psyr_4811 is from the AraC family of transcriptional regulators that have three main regulatory functions: carbon metabolism, stress response and pathogenesis (Gallegos et al., 1997). Up-regulated LysR-type TF CysB controls the biosynthesis of cysteine and sulfur utilization in *E. coli*. (Jovanovic et al., 2003). In *Pseudomonas aeruginosa*, CysB was shown to regulate transcription of the alternative sigma factor PvdS that controls response to iron starvation, virulence, and biogenesis of the pyoverdine siderophores and synthesis of extracellular factors (Imperi et al., 2010). Helix-turn-helix FIS-type TF (Psyr_1736) was up-regulated although qPCR analysis did not confirm RNA-seq data on this gene. Up-regulation of FIS-type TF might suggest ribosomal activity such as transcription of ribosomal RNA and activation of rRNA promoters (Bartlett et al., 2000).

**Table 2:** **Transcription factors differentially expressed in the VBNC cells**.

**Gene ID**	**Log_2_ fold change**	**Annotation**
Psyr_1736	2.89	Helix-turn-helix Fis-type
Psyr_2077	1.65	Transcriptional regulator CysB (LysR)
Psyr_4811	1.44	Transcriptional regulator AraC family
Psyr_3022	1.36	Regulatory protein AsnC/Lrp
Psyr_3102	1.32	Regulatory protein LysR
Psyr_2729	1.29	Regulatory protein, MarR
Psyr_3917	1.26	Regulatory protein LysR
Psyr_3057	1.15	Regulatory protein DeoR
Psyr_0820	1.14	Transcriptional regulator FruR
Psyr_1780	1.13	Regulatory protein TetR
Psyr_3521	1.06	Regulatory protein LysR
Psyr_1386	−1.02	Regulatory protein LysR
Psyr_1702	−1.04	Regulatory protein LuxR
Psyr_4335	−1.05	Regulatory proteins, AsnC/Lrp
Psyr_2536	−1.06	Regulatory protein LysR
Psyr_2183	−1.08	Transcriptional regulator GntR
Psyr_2395	−1.15	Regulatory protein LysR
Psyr_0660	−1.20	Regulatory protein ArsR
Psyr_2607	−1.32	Regulatory protein LuxR
Psyr_3118	−1.39	Transcriptional regulator GntR
Psyr_2250	−1.41	Transcriptional regulator GntR
Psyr_3538	−1.43	Regulatory protein TetR
Psyr_5015	−1.50	Regulatory protein LysR
Psyr_1190	−1.52	Type III transcriptional regulator HrpR
Psyr_1800	−1.71	Regulatory protein LysR
Psyr_3526	−1.76	Regulatory protein LysR
Psyr_3073	−1.87	Regulatory protein LysR
Psyr_2927	−1.90	Regulatory protein, TetR
Psyr_2084	−1.90	Transcriptional regulator GntR
Psyr_2045	−1.93	Regulatory protein LuxR
Psyr_3530	−2.02	Transcriptional regulator GntR
Psyr_2114	−2.12	Response regulator receiver LuxR
Psyr_3347	−2.21	Regulatory protein TetR
Psyr_2920	−2.66	Transcriptional regulator GntR
Psyr_4605	−2.87	Transcriptional regulator PrtN
Psyr_2713	−2.87	Regulatory protein LysR
Psyr_3135	−2.91	Transcriptional regulator GntR
Psyr_1802	−3.14	Regulatory proteins AsnC/Lrp
Psyr_1858	−3.15	Regulatory protein LuxR
Psyr_2336	−3.99	Regulatory proteins IclR

## Discussion

In nature, bacteria are exposed to a wide range of exogenous stress factors, which trigger various survival strategies. The VBNC state appears to be one of these strategies adopted by non-sporulating bacteria. Although it has been described for a number of plant pathogenic bacteria, including *Agrobacterium tumefaciens, Ervinia amylovora, Pseudomonas syringae, Ralstonia solanacearum, Xanthomonas campestris, Xanthomonas axonopodis*, and other species (Wilson and Lindow, 1992; Khan et al., 2010; Oliver, 2010; Kong et al., 2014; Santander et al., 2014), to our knowledge, no studies were conducted on global gene expression profiling of VBNC in plant pathogenic bacteria. Meanwhile, understanding the genetic basis of VBNC state can help to gain valuable insights into molecular mechanisms of bacterial persistence.

In this work, applying RNA-sequencing technology, gene expression changes were profiled in the VBNC state of *P. syringae*, a widely spread plant pathogen virulent in many species of crop plants as well as in non-agricultural niches (Morris et al., 2013). *P. syringae* pathovar *syringae* used in this study, is an opportunistic strain, attacking a variety of plants, including alfalfa, the fourth most widely grown crop in the US. It was recently reported at the 2014 meeting of the American Phytopathological Society that yield losses from bacterial stem blight of alfalfa caused by *P. syringae* pv. *syringae* ALF3 may reach up to 50% of the first harvest (Samac et al., 2014). One of our objectives is to identify genes that control high capacity to tolerate biotic stresses in alfalfa. However, while the plant resources are designated toward defense responses, the pathogen must be capable of creating a suitable environment for its own growth, reproduction and survival. In other words, both the plant and pathogen continuously evolved to bring together a set of genes that enables this interaction (Boyd et al., 2013). Therefore, characterizing the expression of genes, employed by bacteria to circumvent environmental stresses and host defenses is one of the ways to fill critical gaps in our understanding of plant-pathogen interactions.

We have recently developed an *in vitro* model that imitates redox pulse in plant suspension cells inoculated with *P. s*. pv. *syringae* 61 (Mock et al., 2015). The system is based on the *in vitro* oxidation of acetosyringone, a natural metabolite found in the apoplast. Oxidation of acetosyringone prevented multiplication of bacterial cells and caused loss of their culturability (Mock et al., 2015). Fluorescent live/dead staining of bacteria treated with acetosyringone demonstrated that cell death did not occur and measurements of oxygen uptake indicated that they were able to respire (Mock et al., 2015). Although attempts to resuscitate the non-culturable cells were not successful, when added to tobacco cell suspensions, they induced higher levels of extracellular phenolics, normally produced in plants during bacterial infection. We concluded that oxidation of acetosyringone *in vitro* rendered *P. s*. pv. *syringae* into a VBNC-like state (Mock et al., 2015).

Among many questions that emerged from that study (Mock et al., 2015) investigating whether potential ability of the VBNC cells to exhibit continuous gene expression was one of the most interesting. Genes remaining active in VBNC cells are likely to be essential for the occurrence of the state as well as for the resuscitation from the non-culturability under appropriate conditions. In this work, several categories of genes as well as individual genes that may play critical roles in VBNC cells were delineated.

Since the exposure to acetosyringone oxidation was performed on nutrient-deprived bacteria, starvation stress was a possible factor that may have influenced gene expression. However, untreated control bacteria did not render VBNC (Figure 2), meaning that the effect of acetosyringone oxidation on bacterial non-culturability was decisive.

Counts of colony-forming units (CFU) showed that about 1% of bacterial cells survived the treatment and remained in a normal condition i.e., were culturable (Figure 2). While these cells could add some background noise to the observed gene activity, the overall pattern of gene expression shaped by the 99% of the nonculturable bacteria would not change.

By the end of the 3 h the oxidative environment in the reaction mixture was deteriorating due to the high concentration of bacteria scavenging H_2_O_2_ to keep its intracellular concentration low (Figure 1). Differential expression registered at this point involved predominantly genes responsive to oxidative moieties. Since during this oxidation period the vast majority of the bacterial cells (99%) in the population became nonculturable, the same sets of genes could have participated in the triggering and transitioning to the VBNC state.

Overrepresentation of functional categories *Carbohydrate metabolism and transport, QAC metabolism and transport* (quaternary ammonium compounds), *Polyamine metabolisms, Chemosensing & chemotaxis, Energy generation*, and *Peptidoglycan/cell wall polymers* among up-regulated genes accentuates their roles in response to oxidative stress and potentially in the induction of VBNC state. Activation of genes encoding 10 different subunits of NADH dehydrogenase (ubiquinone) in the category *Energy generation* demonstrates the importance of respiratory complex I and NADPH-generating systems in antioxidative defense mechanisms and maintaining redox balance in O_2_-rich environment (Lemire et al., 2010). These nuo genes (of NADH: ubiquinone oxidoreductase) encode proteins in the peripheral arm of the enzyme, including the subunits that bear all known redox groups of complex I and are likely involved in proton translocation, essential for energy consuming processes (Friedrich and Scheide, 2000) (Supplementary Figure 1).

Induction of the gene encoding aconitate hydratase (acnA, Psyr_3404) from the same category (*Energy generation*) might represent an interesting mechanism by which VBNC state is being initiated and maintained. Aconitase is one of the key enzymes in the Krebs citric acid cycle that generates energy by metabolizing carbohydrates, proteins, and fats. Being an iron-sulfur protein, it also performs a completely different function as an iron regulatory protein. The iron-sulfur cluster of the aconitase is unstable and highly sensitive to oxidation by superoxide (Gardner, 2002). When there is not enough iron available in bacterial cell to regenerate the iron-sulfur cluster, the protein binds to mRNA for ferritin, ubiquitous protein that stores and release iron, and inhibits its formation so that less iron is locked up in storage and more released (Goodsell, 2007). Iron has diverse functions in bacterial cells and is required for enzyme activity, secondary metabolism, cell composition, energy production, host-pathogen interactions, etc. Speculatively, during oxidation of acetosyringone in the presense of H_2_O_2_ aconitase shifts to its role as an iron regulatory protein and suspends its action as an essential enzyme in the tricarboxylic acid cycle. Thus, the nutritional requirement for iron could be one of the important characteristics of the transition to VBNC state and aconitase role as a maintenance or survival enzyme during nutritional or oxidative stress may be substantive (http://www.uniprot.org/uniprot/P25516). In plants, aconitase is important for regulating resistance to oxidative stress and cell death (Moeder et al., 2007). Enhanced levels of aconitase in bacteria undergoing oxidative stress due to acetosyringone oxidation *in vitro* or due to the redox pulse *in planta* might be needed to maintain bacterial persistence (Supplementary Figure 2).

Up-regulation of biological functions from the category *Carbohydrate metabolism and transport* that relates to the translocation of nutrients across membrane or efflux of toxins (facilitator transporter Psyr_3138; ABC transporters Psyr_1737 and Psyr_1738; periplasmic binding protein/LacI transcriptional regulator Psyr_3265, Table 1.), suggests adaptation of bacteria to changing environment in order to survive oxidative stress. ABC transporters are important for many different aspects of the bacterial physiology including import of essential nutrients and export of toxic molecules (Davidson et al., 2008). Using the KEGG pathway database (http://www.genome.jp/kegg/pathway.html), activated genes in this category were mapped to the five classes of prokaryotic-type ABC transporters: Mineral and organic ion transporters, Oligosaccharide, polyol and lipid transporters, Monosaccharide transporters, Phosphate and amino acid transporters and Metallic cation, iron siderophore and vitamin B12 transporter (Supplementary Figure 3).

Activation of 13 genes in the Category *Chemosensing & chemotaxis*, most notably Psyr_2356 and Psyr_0905 (methyl-accepting chemotaxis protein, MCP), implies that signaling cascades regulating speed and direction of bacterial motion through the flagellar motor are responding to a complex stress created by ROS and nutrient deprivation (Supplementary Table 1). MCP proteins are embedded in the cytoplasmic membrane and allow bacteria to sensor extracellular matrix, detect nutrients or repellents and orient itself accordingly (Alexander and Zhulin, 2007). KEGG pathway showing the key role of the MCP in transduction of extracellular signals to the downstream proteins in the cytoplasm is presented in Supplementary Figure 4.

Up-regulation of genes in the category *Peptidoglycan/cell wall polymers* points to the changes in the makeup of cell wall polymers in the VBNC cells, particularly peptidoglycan, the main macromolecule involved in cell shape determination and maintenance (Signoretto et al., 2000). Alternations in the biochemical composition of peptidoglycan have been linked not only to the shape of bacteria, but also to growth rate and inhibition of cell division and could aid the induction of the VBNC state (Signoretto et al., 2000). KEGG Peptidoglycan biosynthesis pathway graphically demonstrates the importance of proteins encoded by the activated genes in this biological process (Supplementary Figure 5).

Overrepresentation of the category *Polyamine metabolism and transport* indicates the important role of polyamine homeostasis for VBNC cells. Polyamines are involved in many biological processes in bacteria, such as growth, biosynthesis of siderophores, a diverse array of iron-chelating compounds secreted by bacteria and critical for iron acquisition (Miethke and Marahiel, 2007), stabilization of cell wall and scavenging of free radicals (Wortham et al., 2007).

Significant enrichment of two categories, *Type III secretion system* and *Phytotoxin synthesis and transport*, with down-regulated genes indicates that virulence attributes are not manifested in bacterial cells that are transitioning to or have already entered the VBNC state (Table 1 and Supplementary Figure 6). However, the possibility that repression of pathogenesis-related pathways via crosstalk between signaling systems may be involved in the induction of VBNC, cannot be excluded.

Separate DEGs or clusters of DEGs in many other functional categories which are not overrepresented, may be important for occurrence of VBNC state due to interaction between signaling cascades when they are shared components in different pathways (Supplementary Table 3). Category *Signal transduction mechanisms*, from which six genes were up-regulated, was not overrepresented according to the functional categorization performed as described by Yu et al. (2013) since input genes represented only 15% of the entire category. Genes in this category are part of the phosphorelay signal transduction system, the two-component regulatory system that allows bacteria to adapt to changing environment by quickly modifying cellular physiology, gene expression, cell cycle progression and development (Skerker et al., 2005) (Supplementary Figure 7). Interestingly, that phosphorelay also activates regulators of sporulation in *Bacillus subtilis* (Narula et al., 2012). Quite possibly, gene expression programs resulting in formation of stress-resistant spores in nutrient-deprived bacteria have a certain degree of similarity with those activated during VBNC state as a bacterial survival strategy. Identification of 38 DEGs in another not overrepresented category, *Amino acid metabolism and transport*, demonstrates importance of the amino acid metabolism in the VBNC pathways (Supplementary Table 3 and Supplementary Figure 8). Amino acids are important growth substrates in bacteria and control of their biosynthesis and degradation might be necessary for maintaining the carbon–nitrogen balance in the VBNC cells. Several up-regulated genes in the category *Stress resistance* point to potential involvement of their protein products in the VBNC condition. Among them are other members of the two-component signal transduction system, response regulator receiver Psyr_3709 and sensor histidine kinase Psyr_3708. Out of 13 DEGs in the category *Replication and DNA repair four* genes were up-regulated. Thus, DNA damage due to oxidation of acetosyringone is addressed in the VBNC cells (Supplementary Figure 9). Up-regulation of the genes of the Multidrug resistance, efflux pump MexEF-OprN operon (Psyr_2967, Psyr_2968, and Psyr_69) suggests that the detoxification mechanism in VBNC bacteria is actively exporting harmful substances thus allowing bacteria to survive the stress (Piddock, 2006).

KEGG pathways associated with DEGs identified in this work are shown in the Supplementary Table 4.

Up-regulated transcription factors are likely to have critical functions in transition and maintenance of the VBNC state. Between 11 induced TFs, activation of one gene encoding MarR protein and four LysR genes looks particularly interesting because of their physiological roles as sensors of changing environments. MarR proteins control genes involved in degradation of toxic environmental compounds (including phenolics), virulence, export of harmful chemicals, and resistance to oxidative stress (Grove, 2013). LysR is an ortholog of OxyR in *E. coli*, a transcription factor that is sensitive to oxidation and activates the expression of antioxidant genes in response to hydrogen peroxide in *E. coli* (Zheng et al., 1998). It is reduced in the absence of stress (Zheng et al., 1998). A member of another widely distributed family of TFs, Lrp/AsnC, was up-regulated (Psyr_3022). Lrp/AsnC proteins are ubiquitous among bacteria and specifically regulate amino acid metabolism-related genes in response to environmental signals, such as an abundant nutrient supply or nutrient deprivation (Brinkman et al., 2003; Deng et al., 2011). However, basal expression of another gene encoding protein from the same Lrp/AsnC family (Psyr_1802) was decreased ~14000 fold in not treated, nutrient-deprived bacteria and further decreased in the VBNC cells according to the RNA-seq data (Figure 5 and Supplementary Table 1). This implies a fine-tuned regulation of amino acid metabolism during transition to the VBNC state. Maintaining expression of some TFs at higher levels may be crucial for normal cell division and metabolism, so that when their expression drops dramatically bacteria might enter the VBNC state.

It is unclear whether the reduction of genic antisense RNAs in the VBNC libraries has biological meaning or not. Since transcriptional intervention is one of the proposed regulatory mechanisms by which antisense expression can affect transcription of the messenger RNA (Faghihi and Wahlestedt, 2009; Pelechano and Steinmetz, 2013), decrease in the amount of genic antisense RNAs in the VBNC libraries in theory could be associated with attenuation of transcriptional interference directed toward specific mRNA transcripts needed for transition to VBNC state.

**Figure 7:**
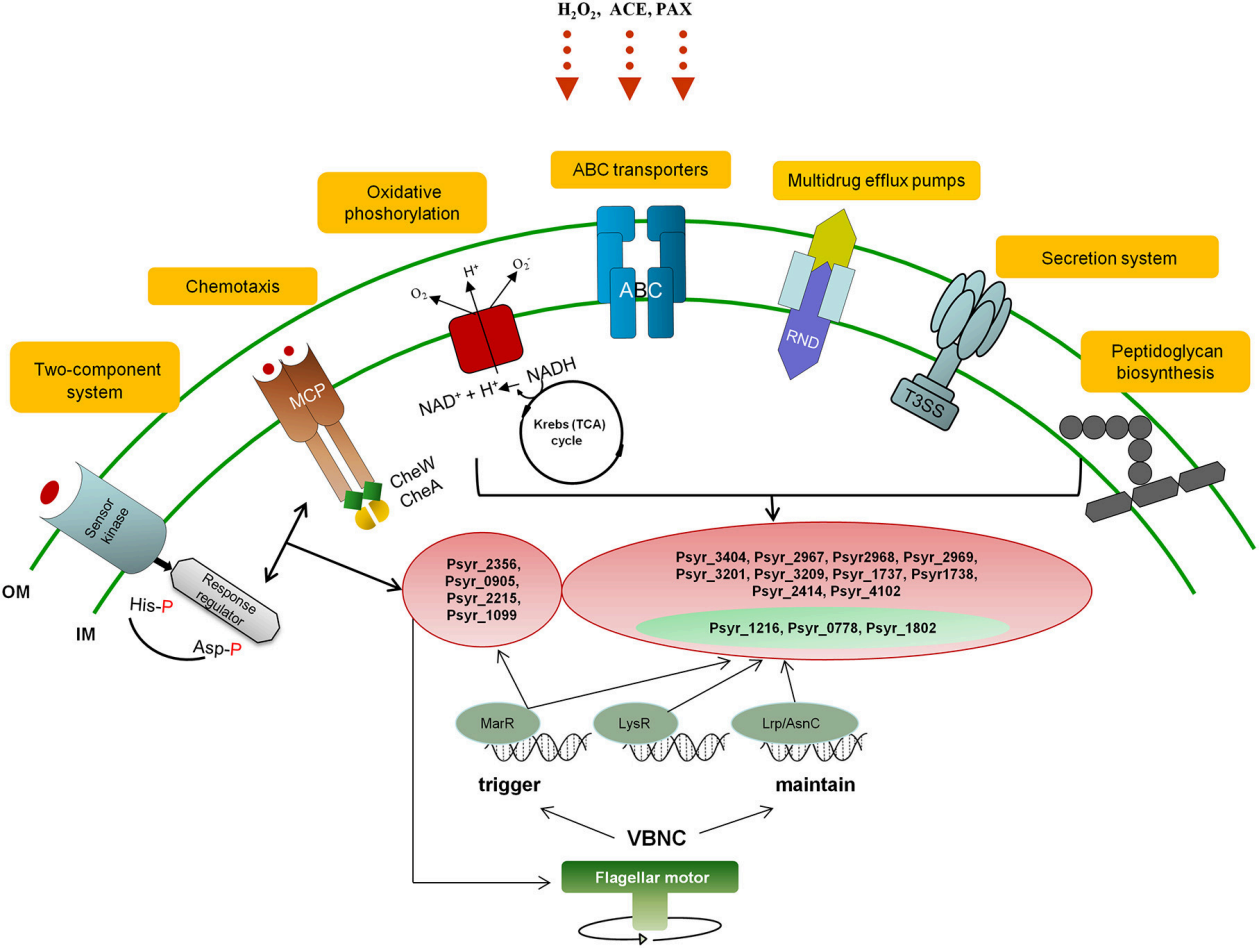
**A diagrammic representation of the proposed network of biochemical reactions that might trigger and maintain VBNC state in ***P. syringae*** pv. ***syringae*****. OM, outer membrane; IM, inner membrane; VBNC, viable but not culturable; ACE, acetosyringone; POX, peroxidase; MCP, Methyl-accepting chemotaxis protein; NADH, Nicotinamide adenine dinucleotide hydrite; ABC, ATP-binding cassette transporter system; T3SS, type III secretion system; RND, resistance/nodulation/division family representing Multidrug resistance (MDR) efflux pumps; MarR, LysR, and Lrp/AsnC transcription factors. Highlighted in red, representative up-regulated genes; highlighted in green, representative down-regulated genes.

We attempted to incorporate predominant KEGG metabolic pathways identified as a result of differential gene expression analysis in the VBNC cells (Oxidative phosphorylation, ABC transporters, Peptidoglycan biosynthesis, Kreb's cycle, Bacterial chemotaxis, Two-component system, Bacterial secretion system), into a network of biochemical reactions that might trigger and maintain VBNC state in *P. syringae* pv. *syringae* (Figure 7).

Initiation of the VBNC state is likely to start with metabolic pathways controlling mechanisms of stimulus-response and movement of bacteria according to the presence of chemicals in the environment (Two-component system and Bacterial chemotaxis, respectively, governed by MarR TF). Bacteria sense stressors through membrane-bound histidine-kinase and response-regulator mediates cellular response via differential expression of target genes (Mascher et al., 2006). For instance, it has been demonstrated that *E. coli* could not enter the VBNC under the stress of osmolarity, pH and starvation state in the absence of the product of sensor kinase (Ramamurthy et al., 2014). Changes in the bacterial motile behavior due to the interplay between adaptively modified chemoreceptors (MCP protein) and motor control may further contribute to triggering of the VBNC mode. Reduction of energy expenditure to minimal levels is one of the key characteristics of VBNC state. Induction of genes encoding subunits of the proton-pumping NADH:ubiquinone oxidoreductase shows that this enzyme is essential for energy consuming processes in the VBNC cells just like it is under the normal conditions (Friedrich and Scheide, 2000). We think that at this stage the bacterial cells have already entered VBNC state in response to environmental change and activity of the respiratory complex I and NADPH-generating systems is critical for maintenance of the cells viability. Selective permeability to nutrients and metabolites provided by ABC transporters can be a prerequisite for VBNC state as well. Survival of the bacterial cells might be further maintained by the Multidrug resistance efflux pump that could confer resistance of the VBNC cells to oxidative moieties. Repression of the pathogenesis-related pathways such as Type III Secretion System (T3SS) may be an additional requirement and/or consequence of the VBNC state, same as down-regulation of genes encoding proteins of the Type VI Secretion System. T6SS mediates interbacterial interactions and is part of the complex molecular machine that delivers effectors to target cells and to other bacteria (Russell et al., 2014). Repression of the T6SS pathway may be attributed to decreased bacterial competition and possible fitness advantage to the VBNC bacteria. Up-regulation of genes involved in peptidoglycan biosynthesis appears to be one of the characteristics of the VBNC cells and may be needed to create a harder wall than that of dividing cells (Signoretto et al., 2000). Maintaining of the VBNC mode is presumably under control of LysR and Lrp/AsnC TFs with suggestive MarR engagement.

In summary, *in vitro* oxidation of acetosyringone by hydrogen peroxide and peroxidase caused *P. s*. pv. *syringae* to enter VBNC mode. Occurrence of the VBNC state was confirmed by CFU counts, visual comparison of culture plates, fluorescent live/dead staining and gene expression analysis. Global gene expression profiling of the VBNC cells identified biological processes presumably associated with this condition in *P. s*. pv. *syringae*. The findings offer insight into what might happen in the plant when bacterial pathogens are first encountered and host defense responses are triggered.

## Author contributions

LN and CB conceived the research. OP, CB, NM, and LN performed experiments. OP and JS performed bioinformatics analysis. LN wrote the manuscript, CB, OP, and JS edited the manuscript. All authors discussed the results and implications and commented on the manuscript at all stages.

### Conflict of interest statement

The authors declare that the research was conducted in the absence of any commercial or financial relationships that could be construed as a potential conflict of interest.
