# Supplementary figures and images for: Gene Expression Profiling in Viable but Nonculturable (VBNC) Cells of Pseudomonas syringae pv. syringae
Source: Front Microbiol. 2015 Dec 18;6:1419. doi: 10.3389/fmicb.2015.01419 (PMC4683178; doi:10.3389/fmicb.2015.01419)

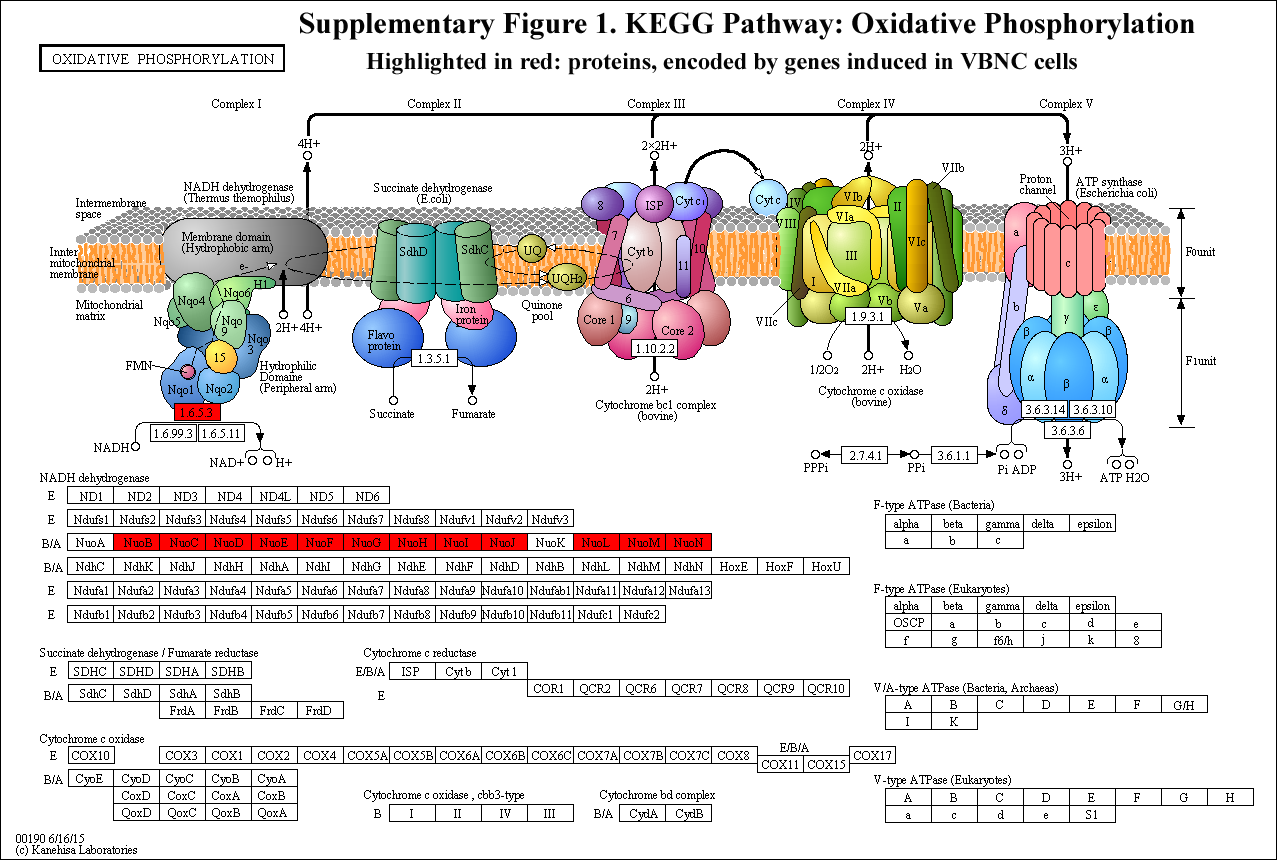

Supplement: Supplementary file 5 [file Image1.PNG]

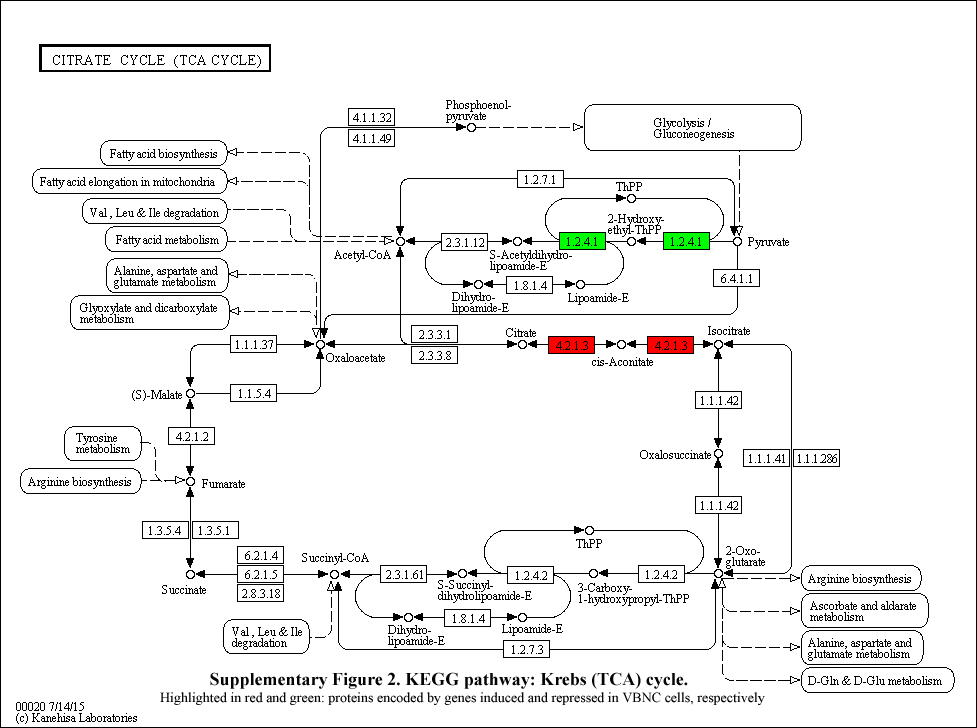

Supplement: Supplementary file 6 [file Image2.PNG]

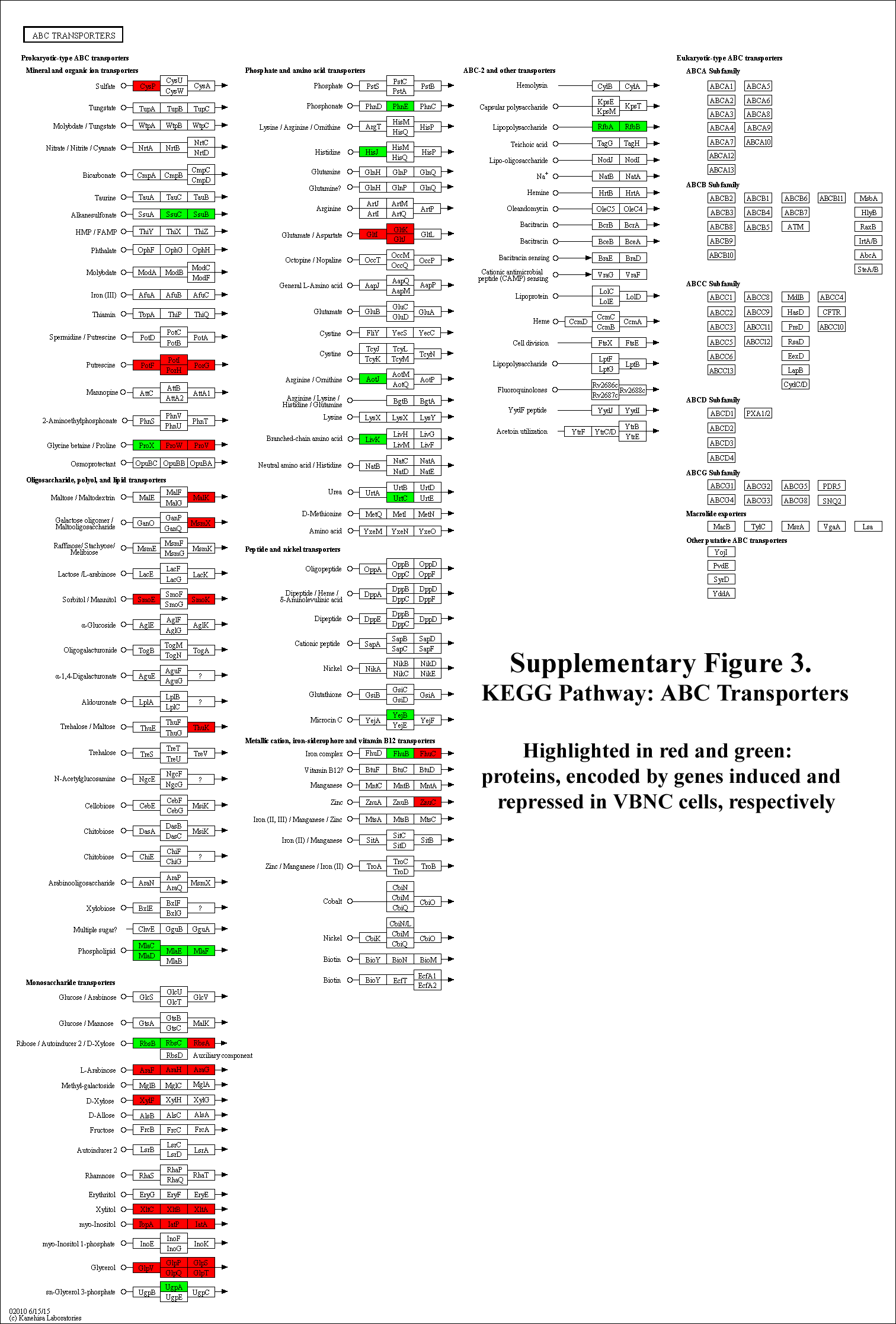

Supplement: Supplementary file 7 [file Image3.PNG]

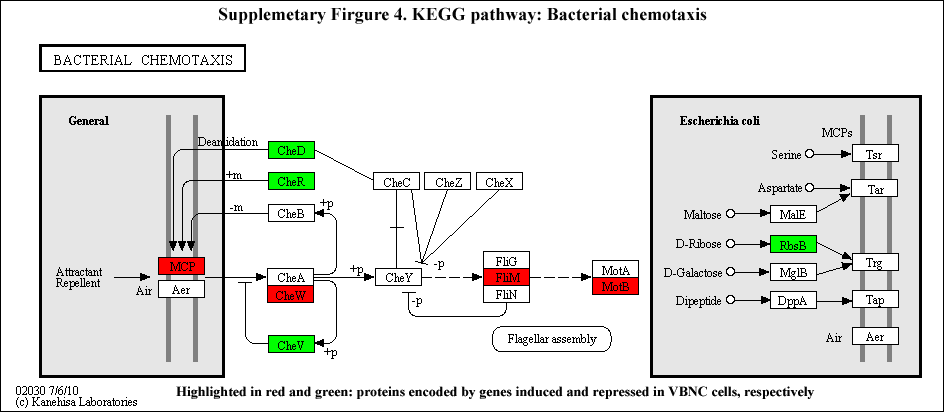

Supplement: Supplementary file 8 [file Image4.PNG]

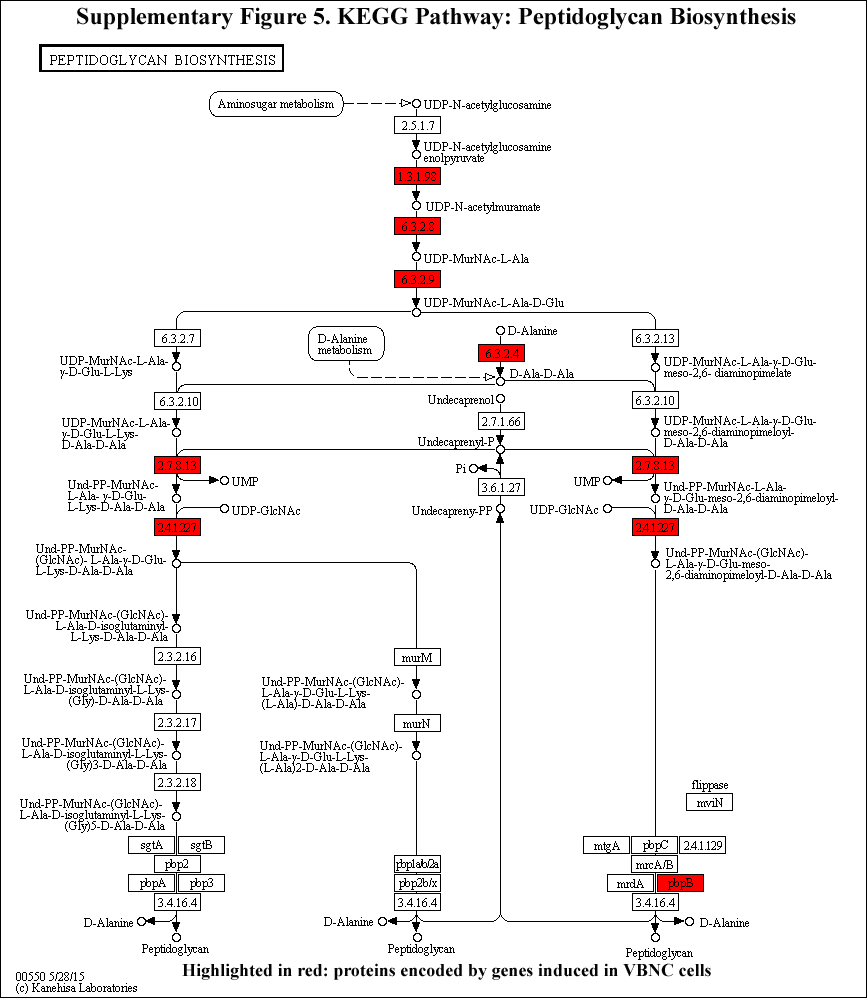

Supplement: Supplementary file 9 [file Image5.PNG]

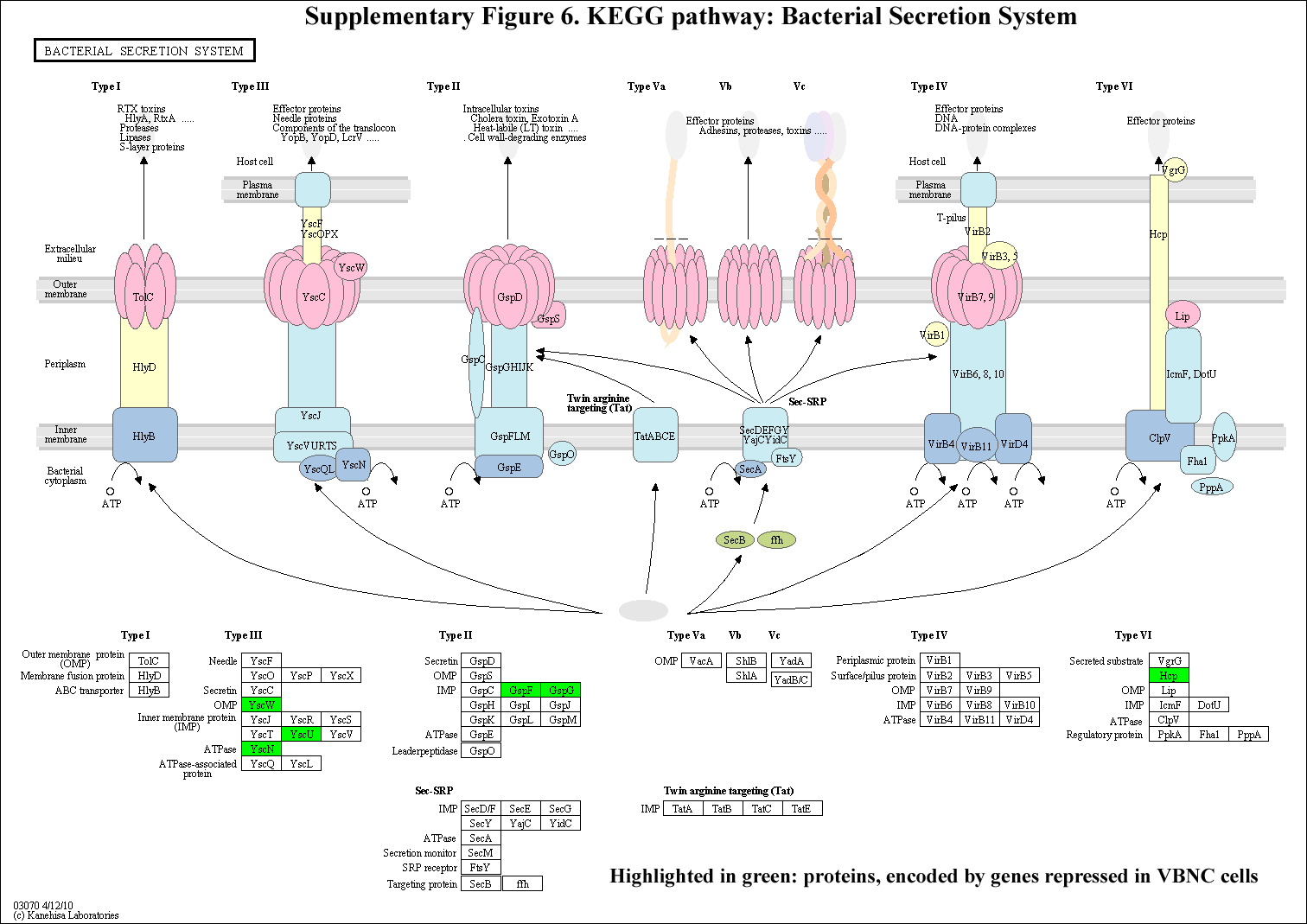

Supplement: Supplementary file 10 [file Image6.PNG]

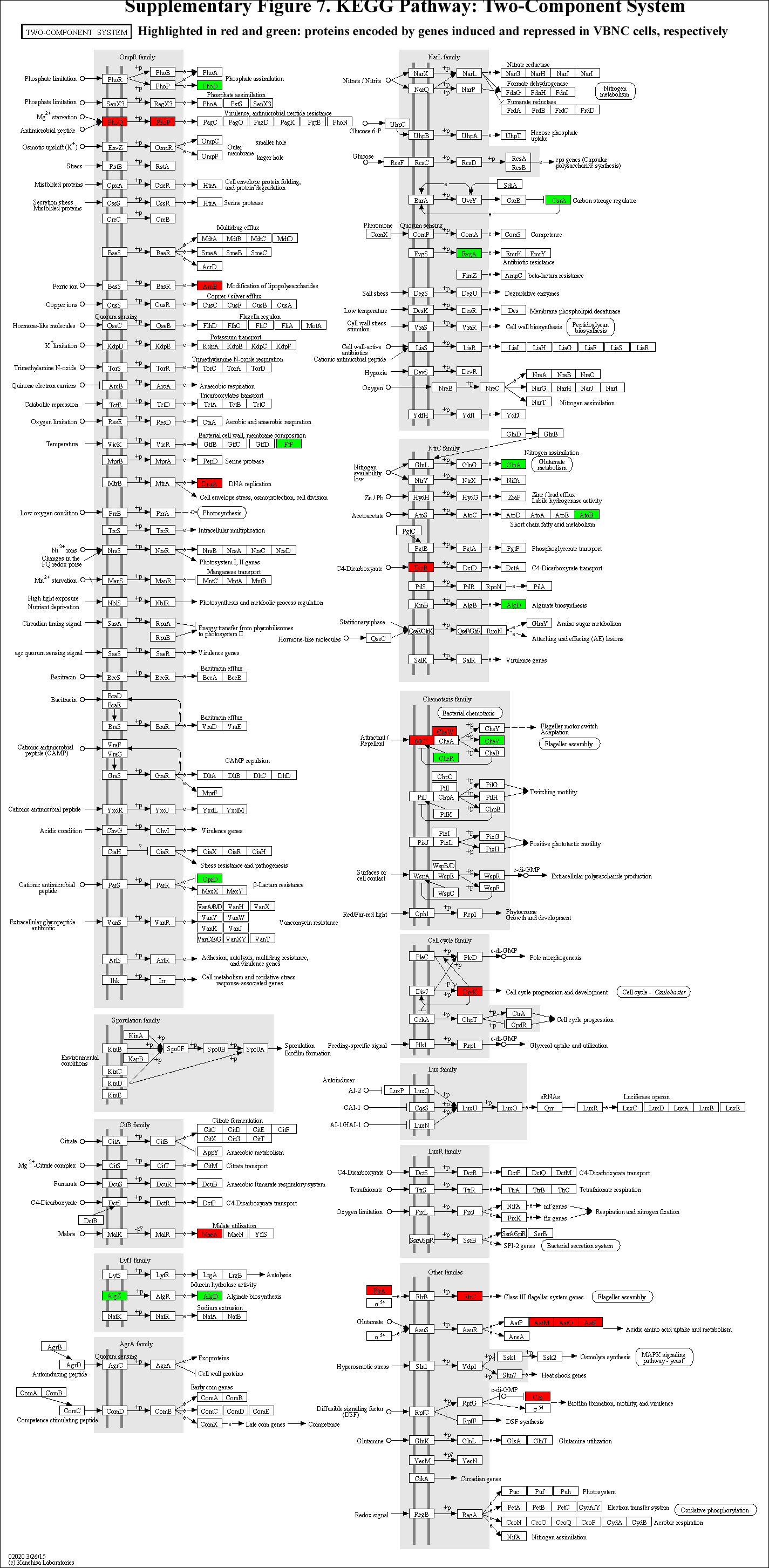

Supplement: Supplementary file 11 [file Image7.PNG]

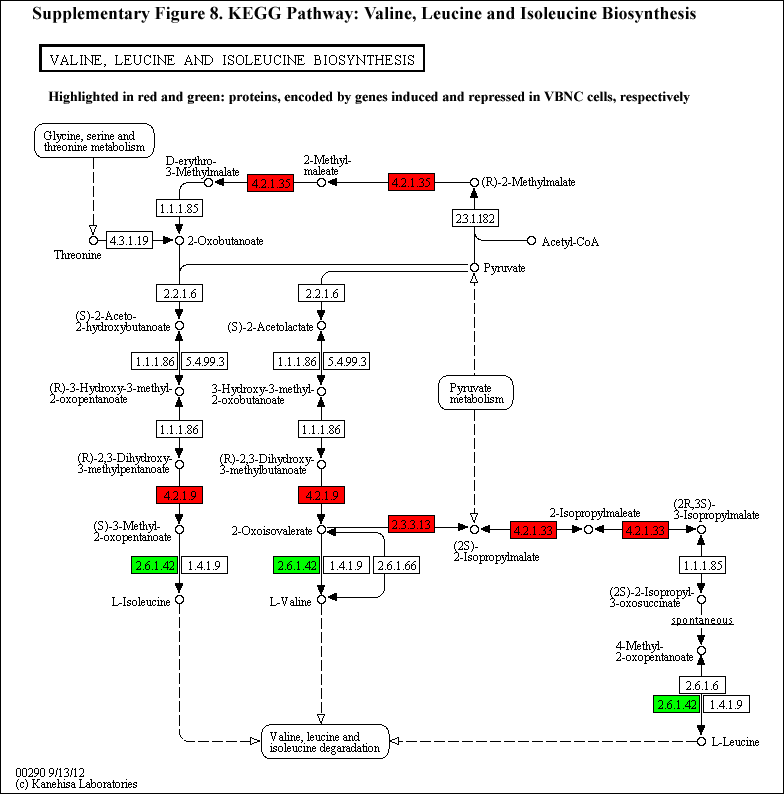

Supplement: Supplementary file 12 [file Image8.PNG]

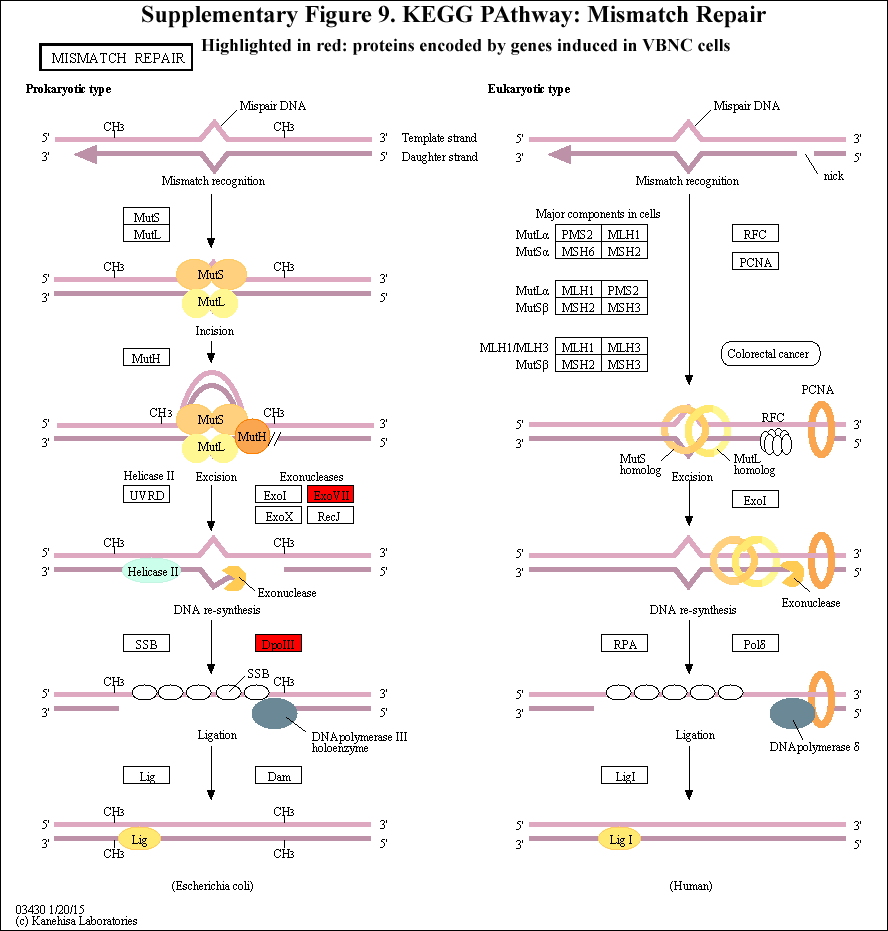

Supplement: Supplementary file 13 [file Image9.PNG]
